# Non-typhoidal *Salmonella* causing urinary tract infection in a young male with renal calculi – a case report and comprehensive review

**DOI:** 10.1099/acmi.0.000610.v5

**Published:** 2023-12-06

**Authors:** Lavanya Sriramajayam, Krishna Mohan Boopathy Vijayaraghavan, Boppe Appalaraju, Sankarganesh Jeyaraj

**Affiliations:** ^1^​ Department of Microbiology, PSG Institute of Medical Sciences and Research, Coimbatore, India; ^2^​ Department of Urology, PSG Institute of Medical Sciences and Research, Coimbatore, India; ^3^​ PSG Center for Molecular Medicine and Therapeutics, PSG Institute of Medical Sciences and Research, Coimbatore, India; ^4^​ PSG Center for Genetics and Molecular Biology, Off Avinashi Road, Coimbatore, India

**Keywords:** non typhoidal salmonella, urinary tract infection, phimosis, *Salmonella enteritidis*

## Abstract

**Introduction.:**

Non-typhoidal Salmonella (NTS) causes urinary tract infections infrequently and are usually associated with presence of genitourinary abnormalities.

**Case presentation.:**

We report a case of immunocompetent male in his early 20 s with phimosis presented with history of dysuria and burning micturition for 4 months. A renal ultrasonography showed presence of bilateral intrarenal calculi. Urine analysis revealed presence of non-typhoidal Salmonella. Automated identification systems performed poorly in identification of serotype. On serotyping, it was identified as *Salmonella enteritidis* in the referral centre. The patient was managed with oral antibiotics.

**Conclusion.:**

This report highlights the issues of inaccurate identification of NTS even with advanced automated systems and early initiation of therapy based on the knowledge of local susceptibility patterns. UTI in immunocompetent individuals by non-typhoidal Salmonella should always be investigated further to rule out genitourinary abnormalities and appropriate antibiotics must be started to avoid chronicity and complications.

## Data Summary

No particular data was generated in this study.

## Introduction

Salmonella is associated with various infections in humans, which includes typhoidal illness and non-typhoidal salmonellosis (NTS) [1]. Non-typhoidal salmonellosis is a disease of great public health importance. Urinary tract infection due to NTS occurs infrequently and is observed predominantly in elderly patients with underlying diseases, diabetes mellitus, urologic abnormalities and immunosuppression. Modes of infection includes hematogenous spread from gastroenteritis and through direct urethral invasion especially in women [2]. *Salmonella enteritidis* is the most frequently isolated Salmonella species in Europe and the USA [3]. Because of absence of an effective nationwide surveillance system, NTS disease burden has been underestimated in India [1]. Here, we present a case of young male with phimosis, renal calculi and chronic urinary tract infection due to non-typhoidal Salmonella after obtaining appropriate ethical approval.

## Case presentation

An unmarried male in his early 20s presented to the outpatient department (OPD) with complaints of dysuria and burning sensation during micturition for the past 4 months. He also had a history of nocturia (2–3 times). He denied history of fever, abdominal pain, increased frequency or urgency of urination, vomiting, hematuria and urethral discharge. This patient has received a course of intravenous antibiotic administration for treatment of UTI in another medical facility and documented evidence of therapy was not available with the patient. He did not have any co-morbidities like diabetes, hypertension or any other past chronic illness. He was a non-smoker and non-alcoholic. Urological history for voiding and storage symptoms was elicited. Apart from presenting symptoms, he did not have any complaints of incomplete voiding, increased intermittency, straining during voiding or urgency incontinence. He did not have any previous history of surgery. He was not on any medications.

On physical examination, he was conscious, oriented and had a normal build. There was no evidence of pallor, icterus, cyanosis, clubbing, lymphadenopathy or pedal oedema. Pulse rate was 88 beats per minute and blood pressure was 116/64 mm Hg. Respiratory rate was normal and auscultation revealed normal breath sounds present. On palpation, abdomen was soft without any tenderness or organomegaly and on auscultation, normal bowel sounds were heard. Physical examination of the cardiovascular and central nervous systems was unremarkable. Examination of genitourinary system revealed presence of congenital phimosis. There was no costovertebral angle tenderness. Digital rectal examination was normal.

### Diagnostic assessments

The patient was advised to undergo ultrasonography (USG) of urinary system and specific laboratory investigations were done. An ultrasonography of urinary system showed right kidney measures 9.9×4.5 cm, normal in size and shows normal parenchymal echoes. Cortico medullary differentiation was present. Pelvicalyceal system and ureter were minimally dilated. Presence of multiple calculi was noticed in all the calyces, largest measuring 9 mm in the lower calyx of right kidney. Left kidney measures 10.6×5.6 cm, normal in size and showed normal parenchymal echoes and cortico medullary differentiation was present. Pelvicalyceal system and ureter were dilated with a calculus in the upper ureter measuring 9 mm. Also a calculus measuring 9 mm was noted in the middle calyx. No free fluid was seen in abdomen. Para aortic region and urinary bladder appeared normal. Prostate appeared normal in size and echoes.

Impression of bilateral intrarenal calculi with right minimal hydroureteronephrosis and left hydroureteronephrosis due to upper ureteric calculus was made.

Serum creatinine and random blood sugar were normal, 0.92 mg dl^−1^ and 123 mg dl^−1^ respectively. Glycosilated HbA1C was 5.4 %. Complete hemogram showed red cell count, total leucocyte count and platelet counts were within normal limits.

Urinalysis revealed macroscopic parameters within normal limits. Microscopy revealed increased WBCs (38 cells per high power field) and chemical analysis also showed the presence of leucocytes (3+), indication of UTI. Sample was negative for crystals or casts.

A clean catch midstream urine sample was sent to the microbiology department for aerobic culture and antimicrobial susceptibility testing. Wet mount microscopic examination showed 3–4 pus cells / hpf and presence of bacteria.

Semiquantitiative culture on urochrome agar showed colourless colonies greater than 10^5^ colony forming units per millilitre and Gram-negative bacilli were seen on Gram-stain performed from the colony. Conventional biochemical tests led to presumptive identification of *Salmonella* spp. Biochemical reactions were as follows: Indole – negative, glucose fermented with gas production, lactose and sucrose not fermented, citrate utilised, urease – negative, mannitol motility medium – fermented and motile, triple sugar iron medium – K/A with gas and H_2_S, phenylpyruvic acid test – negative. Serotyping was performed with the antisera procured from Central Research Institute [CRI], Kasauli, India. The isolate was agglutinating with group specific O9 antisera but not with Hd. Denoting that it belonged to O9 group by Kauffman white Classification scheme. The isolate was found susceptible to ampicillin, ceftriaxone, ceftazidime, ciprofloxacin, azithromycin, chloramphenicol and cotrimoxazole by Kirby–Bauer disc diffusion method (CLSI). The isolate was identified as *Salmonella enterica* Group B or Group D with 98 % probability using Vitek two automated identification system (bioMérieux SA, France). The isolate was also subjected to MALDI-TOF Biotyper (Bruker Daltonik GmbH, Bremen, Germany) and was identified as *Salmonella cholerasuis* with a score value of 2.33, indicating highly probable species identification. This isolate was further sent to the National Salmonella and Escherichia Centre (NSEC), National Reference Centre for serotyping of *Salmonella* and *Escherichia coli *, CRI Kasauli, India and it was identified as *Salmonella enteritidis* (antigenic structure – 9,12;gm:-) using serovar typing method.

### Therapeutic interventions

The patient was started on T.ciprofloxacin 500 mg twice daily (BD) for 14 days for the treatment of UTI. He was also advised surgery (bilateral ureterorenoscopy [URS] with laser lithotripsy with bilateral Double J [DJ] stenting) for renal calculi and circumcision was advised for phimosis.

### Follow-up and outcome

On telephonic reminder post-two weeks of his visit to OPD, for a review visit, he informed that his symptoms had resolved. Unfortunately the patient was lost to follow up and surgery could not be performed at our centre.

## Discussion

*Salmonella enteritidis* is emerging as a major cause of food-borne diseases and usually is associated with chicken eggs [4, 5]. Though extra-gastrointestinal tract focal infection has been mentioned, UTI due to NTS is uncommon. Worldwide, the overall incidence of NTS positive urine cultures has been estimated to be between 0.015 and 0.118% and about 18 % of these patients become chronic urinary carriers [6]. In India, *Salmonella enteritidis* along with *Salmonella typhimurium* have been reported to be amongst the most common causes of human salmonellosis, which were found to account for 57–67 % of the total Salmonella isolates which were obtained from various human samples (includes blood, pus, faeces, urine and tissue) [1, 4]. NTS UTI is a surrogate marker of underlying predisposing factors such as unrecognized immune system suppression or anatomical abnormalities of genitourinary tract [2]. Eng *et al*. published a case report along with a literature review of published reports containing data from 29 patients with UTI and NTS before the year 1987. Six cases out of 29 were found to be with urological abnormalities [7]. In a comprehensive literature review published by Cohen *et al*., the authors reported 41/54 cases were carrying NTS where 18 cases were having urological abnormalities including renal calculi [8]. In a retrospective analysis study published in 1996, Ramos *et al*. showed out of 28 cases with bacteriuria 24 patients were found to be infected with *Salmonella enteritidis *. Fourteen out of 28 patients were found to be with urological abnormalities [9]. In a case reports study describing the retrospective analysis of 19 cases with UTI and NTS in University Hospital of Guadalajara, Spain, it has been shown that chronic diseases were found in 14 patients, eight patients had urolological abnormalities and *S. enteritidis* was the most common pathogen detected in 16 cases [10]. It has been shown that *Salmonella enterica* serovar Typhi has the ability to form biofilms on the surface of human gall stones, likewise, NTS might colonize in the urinary tract system and form biofilms [11].

UTI leads to the formation of kidney stones where bacteria are responsible for the formation of renal calculi through urease enzymes. In a 2012, prospective study conducted by Tavichakorntrakool *et al*., the authors have shown that the bacterial cultures isolated from renal calculi from 100 stone former patients remained the same in both the core and the peripheral parts of the calculi. Renal calculi were associated with salmonella infection in only one patient of all the cases analysed [12]. *Salmonella enteritidis* infection of the urinary tract is an uncommon condition. In phimosis condition, bacterial colonization occurring in the inner region of prepuce could lead to the establishment of urinary infections and circumcision plays an important role in minimising the risk of UTI [13]. Results from non-randomized studies showed febrile UTI has been decreased in circumcised males compared to non-circumcised group [14]. A meta analysis study published in 2005, included one randomized controlled trial, four cohort studies and seven case-control studies for the analysis with results showing the beneficial effect of circumcision in males who have a high risk of UTI. It should be noted that, the prevalence of circumcised males in countries like India, China, Japan and Taiwan is less than 20 % [15]. Recently, it has been shown that boys with retractable prepuce had a lower risk of UTI when compared to cases with high grade phimosis and non-retractable foreskin. Boys with high grade phimosis (4–5) were prone to develop UTI when compared to the cases with lower grade phimosis (0–3) [16]. In this study, our patient had phimosis, this further emphasizes that a thorough clinical examination of the genitourinary system is mandatory especially when the predisposition to chronic UTI is unlikely.

From literature review, it is understood that the pathogenesis of UTI by NTS is either by direct urethral invasion or by hematogenous spread in patients with previous episodes of gastroenteritis [2, 17]. Our patient did not have any previous history of gastroenteritis. Rodrigues *et al*. reported 85 % of patients with Salmonella cystitis had concurrent diarrhoea, whereas all patients with Salmonella pyelonephritis had concurrent bacteraemia in their study [18]. Most recent case series report patients with only urinary tract symptoms and Salmonella isolated in urine culture but negative stool cultures for Salmonella species [19].

Salmonella serotype groups C1 and E were more commonly associated with UTIs in a study including 799 isolates of NTs from urine samples, in contrast to group D Salmonella UTI, as described in our case [20]. Identification of NTS requires antisera to various serovars which is usually done at reference centres. More widely available systems for rapid identification of organisms like MALDI-TOF provides high accuracy in identification of Salmonella at species level but is limited to type or subtype Salmonella serovars [21, 22].

NTS urine infection may be difficult to treat and early institution of antibiotics is associated with a favourable outcome. Complications due to Salmonella UTI warrants prolonged treatment, even though recurrences may still be seen [2].

Multidrug resistance (MDR) is not uncommon among NTS and it was observed in 75 % of NTS isolates in Sub-Saharan Africa. The number of ceftriaxone-resistant bloodstream isolates was seen to be 5 % between 2003 and 2013 in the USA [23]. Resistance to ciprofloxacin was noticed as 8.9 % among NTS isolated from eggs. Also high prevalence of multiple antimicrobial resistance among NTS strains in a study in south India suggests possible prior selection by use of antimicrobials in egg production [24]. Thus, sound judgement in antimicrobial use in both humans and food-producing animals is paramount to limit the spread of antibiotic resistance and the emergence of more virulent strains of Salmonella [23]. Though this case report emphasizes thorough genitourinary examination in cases of chronic UTI and prior knowledge of prevalent NTS strains in the given locality, the main limitation of the study is the causal relationship between phimosis, renal calculi, and UTI. Renal calculi are more commonly associated with the development of chronic UTI by other well-known UTI-causing pathogens and rarely with NTS. Another limitation is the need to follow up with the patient for further interventions.

To summarize, *Salmonella enteritidis* is one of the infrequent pathogens causing chronic urinary tract infection and its detection is a challenge in resource limited settings due to inaccessibility to antisera. Automated identification systems have limitations in detection of Salmonellae serotypes. As multidrug resistance among NTS is rising due to inappropriate antibiotic usage in both human and animal industries, the knowledge of prevalence of MDR in local to geographic area is a must before starting therapy.
